# HIV-1 Capture and Transmission by Dendritic Cells: The Role of Viral Glycolipids and the Cellular Receptor Siglec-1

**DOI:** 10.1371/journal.ppat.1004146

**Published:** 2014-07-17

**Authors:** Nuria Izquierdo-Useros, Maier Lorizate, Paul J. McLaren, Amalio Telenti, Hans-Georg Kräusslich, Javier Martinez-Picado

**Affiliations:** 1 AIDS Research Institute IrsiCaixa, Institut d'Investigació en Ciències de la Salut Germans Trias i Pujol, Universitat Autònoma de Barcelona, Badalona, Spain; 2 Unidad de Biofisica (CSIC-UPV/EHU) and Departamento de Bioquímica, Universidad del Pais Vasco, Bilbao, Spain; 3 Institute of Microbiology, University Hospital Center and University of Lausanne, Lausanne, Switzerland; 4 Department of Infectious Diseases, Virology, Universitätsklinikum Heidelberg, Heidelberg, Germany; 5 Universitat de Vic–Universitat Central de Catalunya (UVic-UCC), Vic, Spain; 6 Institució Catalana de Recerca i Estudis Avançats (ICREA), Barcelona, Spain; University of Alberta, Canada

## Abstract

Dendritic cells (DCs) are essential in order to combat invading viruses and trigger antiviral responses. Paradoxically, in the case of HIV-1, DCs might contribute to viral pathogenesis through *trans*-infection, a mechanism that promotes viral capture and transmission to target cells, especially after DC maturation. In this review, we highlight recent evidence identifying sialyllactose-containing gangliosides in the viral membrane and the cellular lectin Siglec-1 as critical determinants for HIV-1 capture and storage by mature DCs and for DC-mediated *trans*-infection of T cells. In contrast, DC-SIGN, long considered to be the main receptor for DC capture of HIV-1, plays a minor role in mature DC-mediated HIV-1 capture and *trans*-infection.

## Introduction

Dendritic cells (DCs) are derived from bone marrow precursors and have a major role in antigen presentation and induction of host immune responses. DCs express a plethora of pathogen recognition receptors, such as toll-like receptors, scavenger receptors, and lectin receptors, which recognize evolutionarily conserved pathogen-associated molecular patterns and contribute to antimicrobial defense. Upon infection, pathogen sensing by immature DCs (iDCs) in mucosal tissues elicits the secretion of cytokines and chemokines. This early innate response creates an inflammatory microenvironment that prompts DC maturation and migration to secondary lymphoid tissues. Concurrently, co-stimulatory molecules are expressed on the cell membrane, preparing DCs for competent T cell priming. In the T cell areas of the lymph node, fully mature DCs (mDCs) present pathogen-derived antigens to T lymphocytes. By these means, DCs coordinate innate and adaptive immune responses against invading pathogens and thus have a critical role in limiting viral infections [1]–[3]. In the course of the HIV-1 infection, however, the contribution of DCs to the antiviral state could be confounded by their ability to facilitate HIV-1 transmission to bystander CD4^+^ T cells and promote viral spread.

Since DCs express the HIV receptor CD4 and viral coreceptors on their surface [4], [5], they are expected to be infected by HIV-1. In cell culture, however, the percentage of HIV-1-infected DCs is always much lower than for activated CD4^+^ T cells [6]–[9] or macrophages. Moreover, large amounts of HIV-1 are required to successfully infect DCs. DC maturation further limits infection: mDCs are 10-fold to 100-fold less susceptible to HIV-1 than iDCs [6], [10], [11]. Thus, although the most important DC subsets are susceptible to HIV-1 infection [12]–[15], this seems to be a rare event. HIV-1 infection of DCs also appears to be uncommon in vivo, although it has been reported for both cutaneous and mucosal DCs [9], [16]. The identification of the host restriction factor SAMHD1 (sterile alpha motif domain– and HD domain–containing protein 1) helped to explain the limited HIV-1 infection of DCs [17], [18]. SAMHD1 restricts infection by reducing the nucleotide pool available for reverse transcription, thereby limiting replication of the viral genome [19].

In contrast to HIV-1, HIV-2 naturally infects DCs [20], and this function depends on counteraction of SAMHD1 by Vpx, a viral protein not present in HIV-1 [17], [18]. Vpx is incorporated into HIV-2 particles and is released after viral fusion, inducing degradation of host cell SAMHD1. However, efficient DC infection is not required for disease progression, since HIV-1 is much more pathogenic than HIV-2. This discrepancy might be explained by differences in innate sensing. HIV-2 genome replication in infected DCs is detected by the innate sensor cGAS, a cyclic guanosine or adenosine monophosphate synthase that recognizes viral DNA and triggers immune responses [20], [21], while SAMHD1-mediated restriction of HIV-1 prevents cytoplasmic cDNA synthesis and consequently precludes induction of antiviral type I interferon responses [20].

Despite low rates of infection by HIV-1, DCs can efficiently capture HIV-1 and mediate potent viral transmission, thus promoting a vigorous infection of CD4^+^ T cells [11] in the absence of productive DC infection [22] or innate immune detection. This so-called *trans*-infection is particularly robust for mDCs [6], [23], [24] and takes place at viral concentrations that do not allow for efficient infection of CD4^+^ T cells by cell-free virus [25]. HIV-1 *trans*-infection involves capture and internalization of intact virions by DCs, trafficking of trapped viruses without membrane fusion, and finally release of infectious virions towards contacting CD4^+^ T cells [25], [26].

Based on their ability to retain virions and travel to lymphoid tissues, it was initially proposed that iDCs act as “Trojan horses,” capturing HIV-1 in the mucosa and then migrating to secondary lymphoid tissues, where stored HIV-1 could be transmitted to CD4^+^ T cells and contribute to the spread of infection [25], [27]. However, the capacity of iDCs to function as “Trojan horses” is limited: iDCs quickly degrade most of the incoming virions, and *trans*-infection by iDCs is thus only possible in the hours that follow a viral encounter [28]. By contrast, viral capture is potently enhanced in mDCs [29], [30], and infectious HIV-1 is stored in an apparently intracellular compartment in these cells. Storage has been reported to occur in a nonclassical endosomal compartment enriched in tetraspanins [31] or at an invagination of the plasma membrane that is distinct from endocytic vesicles [32]. In this compartment, HIV-1 is expected to be protected from endosomal or cytosolic degradation pathways [26]. The precise nature and origin of the capture and storage compartment is currently unknown, and it is also not clear whether it is constitutive or virus induced. An interesting parallel exists with the budding compartment in HIV-infected macrophages, which constitutes an invagination of the plasma membrane that can be rapidly shifted to a T cell contact zone, thus facilitating cell-to-cell transfer of HIV-1 [33], [34]. Kinetic analysis suggests that the HIV-1 capture and storage compartment in mDCs gradually connects with the extracellular milieu and is constantly remodeled [35], which may favor both viral accumulation and subsequent transfer.

HIV-1 transmission has been suggested to occur primarily at a zone of cell-to-cell contact—the infectious synapse—that resembles the immunological synapse, a spatially segregated supramolecular structure formed by T cells to recognize antigens presented by DCs [36]. Upon contact with mDCs, the HIV entry receptors CD4, CCR5, and CXCR4 on CD4^+^ T cells are concentrated in the contact zone [24], thus providing optimal conditions for viral entry. The virus storage compartment in DCs is shifted towards this contact zone, facilitating rapid and efficient infection of the neighboring T cell [24], [31], [37], [38]. Although *trans*-infection via mDCs does not involve new virus production from DCs, this transfer mode appears to be highly related to the virological synapse established between HIV-1 infected cells and target cells [38].

*Trans*-infection of CD4^+^ T cells by HIV-1 captured on mDCs appears to be a particularly potent mechanism of viral transmission and is thus thought to play a major role in HIV-1 spread in lymphoid tissues in vivo. Thus, the capacity of mDCs to capture HIV-1 for *trans*-infection while being largely resistant to HIV-1 infection may be an important aspect of HIV-1 pathogenesis. In this review, we will focus on the role of recently identified viral and cellular factors involved in HIV-1 capture by mDCs and discuss how they might contribute to HIV-1 immune escape and pathogenesis.

## HIV-1 *Trans*-Infection by mDCs: No Sign of DC-SIGN

**Figure 1:**
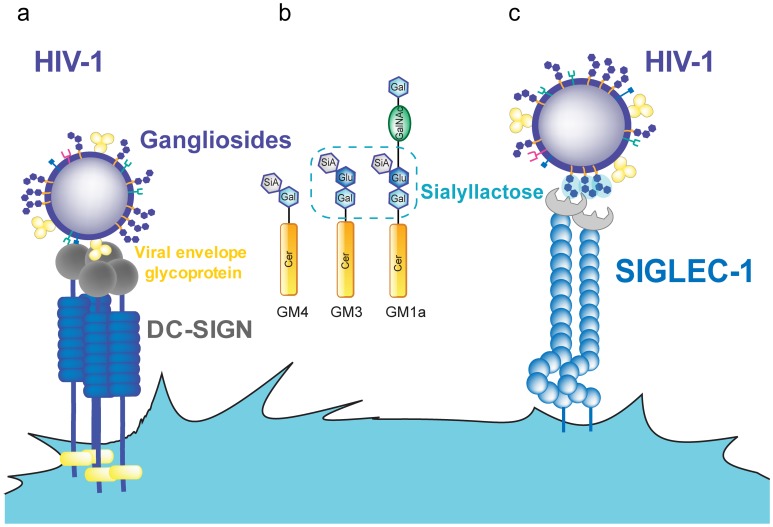
HIV-1 binding to DC receptors. **A.** HIV-1 can bind to DC-SIGN via recognition of the viral envelope glycoprotein. **B.** Several gangliosides in the HIV-1 lipid membrane expose a sialyllactose moiety, while GM4 only carries sialic acid on galactose. **C.** Siglec-1 can capture HIV-1 through recognition of sialyllactose moieties of viral membrane gangliosides. Abbreviations: Cer (ceramide), Gal (galactose), GalNAc (N-acetylgalactosamine), Glu (glucose), SiA (sialic acid).

The dendritic cell-specific intercellular adhesion molecule-3 (ICAM)–grabbing non-integrin (DC-SIGN) has previously been suggested to be the main capture receptor for HIV-1 on DCs [25], [27]. DC-SIGN is a C-type lectin receptor expressed abundantly on the surface of iDCs that interacts with the HIV surface glycoprotein gp120 [25], [39] and thus acts as a capture receptor for HIV-1 on iDCs (Figure 1A). The affinity of gp120 for DC-SIGN is five times greater than for its cognate receptor CD4 [39], suggesting that DC-SIGN on iDCs could be of particular importance when only few HIV-1 particles are present, such as in early infection [25]. This observation led to the “Trojan horse” hypothesis, which argues that DC-SIGN captures HIV-1 in the mucosa and facilitates its transport to secondary lymphoid organs rich in CD4^+^ T cells that can be efficiently *trans*-infected [25]. However, the restricted capacity of iDCs to sustain *trans*-infection [28], [40] and the limited contribution of DC-SIGN to viral transmission reported in several independent studies [29], [30], [41]–[47] argued against the original “Trojan horse” hypothesis.

In contrast to iDCs, mDCs located in lymphoid tissues can effectively transfer HIV-1 to T cells. Cell-associated transfer via the virological synapse is believed to constitute the major mode of transmission in the densely populated lymphoid tissue. The continuous interaction between mDCs and CD4^+^ T cells [48] could be particularly relevant to this tissue, allowing for infectious synapse formation. However, DC-SIGN expression is reduced upon DC maturation [25], [49], [50], while HIV-1 capture and *trans*-infection are potently enhanced [23], [24], [29], [30]. DC-SIGN blocking agents such as mannan or anti-DC-SIGN antibodies have minimal effects on capture and transfer of HIV-1 by mDCs, while they completely abrogate viral capture and transfer in DC-SIGN-transfected cell lines [29], [30]. Furthermore, DC-SIGN is not expressed on the surface of blood myeloid DCs and Langerhans cells [5], [51], while these cells efficiently capture and *trans*-infect HIV-1, especially after maturation [29], [52].

These observations indicate that DC-SIGN is dispensable for HIV-1 capture by mDCs and suggest that this process is mediated by other cell-surface molecules. This hypothesis is strongly supported by the finding that HIV-1 capture by mDCs does not require the viral envelope glycoproteins [29], [53], while DC-SIGN interaction with HIV-1 occurs via gp120 [25]. Mature DCs and blood myeloid DCs capture HIV-1 particles lacking viral envelope glycoproteins as efficiently as wild-type virus, thus excluding gp120-interacting molecules as essential binding receptors [29]. Other HIV-1 receptors described on DCs, including CD4, several C-type lectins (e.g., mannose-binding receptor, dendritic cell immunoreceptor (DCIR), and trypsin-sensitive receptors), and glycosphingolipids (e.g., galactosyl-ceramide) also bind to the envelope glycoproteins [29], [41]–[47] and are therefore also excluded as receptors for capture of particles lacking viral envelope proteins. These combined results indicate that another surface receptor must be responsible for HIV-1 capture and transmission by mDCs and that this receptor should recognize viral membrane constituents other than the HIV-1 envelope glycoprotein.

## Bitter-Sweet Attraction between HIV-1 and mDCs

Since the viral envelope glycoproteins are dispensable for mDC capture, other constituents of the viral membrane should be responsible. These molecules could be proteins, lipids, or sugars but should be widely distributed, because HIV-1 capture by mDCs is independent of the producer cell type [29], [53]. Recognition molecules should also be present in the membrane of cellular microvesicles (e.g., exosomes), which undergo capture by mDCs similar to HIV-1 [54]. Both exosomes and HIV-1 appear to bud from cholesterol-enriched microdomains in the T cell plasma membrane [55]–[57] and share glycosphingolipids and various membrane proteins that reside in lipid rafts. Capture and transfer of HIV-1 by mDCs converges with the exosome trafficking pathway and HIV-1 and exosomes compete for mDC capture, indicating that they utilize the same pathway [54].

Internalization of HIV-1 or exosomes is not abrogated by pretreatment of these particles with proteases; therefore, capture appears to be independent of membrane proteins. In contrast, modifying the lipid composition by specific lipid biosynthesis inhibitors in producer cells affects particle capture by mDCs without altering particle release [53], [54]. Specifically, treatment of HIV-1- or exosome-producing cells with inhibitors of glycosphingolipid biosynthesis yielded particles with reduced glycosphingolipid content, which exhibited reduced capture by mDCs [53], [54]. These findings suggested a critical role of glycosphingolipids for mDC capture and storage of HIV-1 and exosomes [54].

If capture by mDCs is mediated by the particle's lipid composition and independent of membrane proteins, this property should also be observed for liposomes that have the size and lipid composition of HIV-1 but lack any protein. This is indeed the case: liposomes mimicking the lipid composition of HIV-1 were efficiently recognized by mDCs and competed with HIV-1 for mDC capture [58], [59]. Gangliosides represent the predominant group of membrane glycosphingolipids, and mDC capture showed complete ganglioside dependence when comparing liposomes containing or lacking gangliosides. Furthermore, ganglioside-containing liposomes trafficked to the same compartment as HIV-1 and exosomes, and these particles competed with each other for mDC capture [54], [58].

All gangliosides are composed of a ceramide molecule and a variety of sialylated carbohydrate head groups (Figure 1B). Liposomes containing only ceramide were not captured by mDCs, suggesting that the sialylated carbohydrate head group constitutes the molecular recognition domain. Sialic acid on cellular membrane molecules has been identified as an attachment receptor for several pathogens and toxins [60]–[64]. It therefore appeared to be a good candidate for a potential mDC recognition moiety. Removing sialic acid from the membrane of liposomes or viruses by neuraminidase treatment or reconstituting liposomes with asialo-gangliosides abolished capture by mDCs, indicating that sialic acid is necessary for mDC recognition, though not sufficient. No capture was observed for particles containing GM4, the simplest ganglioside (Figure 1B), while mDC capture was efficient when the membrane contained GM1, GM2, or GM3. GM4 has its sialic acid moiety bound to a single galactose, while GM3, the next ganglioside in complexity, as well as GM1 and GM2 carries sialic acid bound to lactose as a head group. This sialyllactose head group (Figure 1B) therefore appears to constitute the molecular determinant for mDC recognition [58]. Accordingly, soluble sialyllactose efficiently prevented HIV-1 capture by mDCs when added at high concentrations. However, efficient particle capture requires membrane gangliosides, and attachment of sialyllactose to ceramide is probably needed for a higher binding avidity. The hydrophilic moiety of ceramide in the membrane interface may be part of the recognition domain, either increasing the binding affinity or orienting the sialyllactose group upon hydrophobic interaction between ceramide and other membrane constituents. Both GM1 and GM3 can serve as mDC recognition molecules when incorporated at high concentrations, while GM3 appears to be more efficient at limiting ganglioside concentrations. Accordingly, knockdown of GM3 (but not GM1) from HIV-1 producer cells and hence from virions strongly reduced capture by mDCs [59].

## Siglec-1 (CD169) Is the Capture Receptor for HIV-1 on mDCs

The observation that the sialyllactose moiety of viral membrane gangliosides is recognized upon HIV-1 capture by mDCs suggested that the attachment receptor may be a sialic-acid-binding cell-surface molecule. Obvious candidates were the family of sialic-acid-binding immunoglobulin-like lectins (Siglecs): these type I transmembrane proteins carry an amino-terminal V-set domain that directly interacts with sialylated ligands, mediating both cellular interactions and immune responses [65]. Using transcriptome analysis, *SIGLEC1* gene (coding for Siglec-1, CD169, or Sialoadhesin) was identified as the only member of the Siglec family that was significantly up-regulated upon DC maturation with lipopolysaccharide (LPS) [66]. Since this treatment had been shown to strongly enhance the capture capacity of mDCs for HIV-1, Siglec-1 was considered a prime candidate for a capture receptor. Similar results were observed upon type I interferon treatment of mDCs, which enhanced both Siglec-1 surface expression and HIV-1 capture [67].

Several lines of evidence demonstrated that Siglec-1 expression correlates with the HIV-1 capture and *trans*-infection capacity of primary DCs [66], [67]. Specific antibodies against Siglec-1 inhibited HIV-1 capture in a dose-dependent manner. In addition, Siglec-1 knockdown by small interfering RNA (siRNA) strongly reduced viral capture and *trans*-infection, while de novo expression of Siglec-1 in cells devoid of this receptor enhanced viral capture and *trans*-infection. Hence, Siglec-1 was identified as a novel DC receptor for HIV-1 capture and *trans*-infection (Figure 1C), which is highly up-regulated in blood myeloid DCs exposed to LPS or type I interferon [66], [67]. In contrast, other members of the Siglec family (i.e., Siglec-5 or Siglec-7) had no effect in these assays despite their capacity to bind sialic acid.

Induction of Siglec-1 expression upon LPS or interferon treatment explains why mDCs are able to capture higher amounts of HIV-1 than iDCs and why this process does not require the viral surface glycoprotein but relies on viral membrane gangliosides (Figure 1C). Siglec-1 recognition has been previously suggested to play a role in enhancing macrophage infection by HIV-1 [68], but this study reported a Siglec-1 interaction with sialylated viral envelope proteins and not with membrane gangliosides. This enhancing effect is likely due to increased viral capture and thus prolonged exposure to the cell-surface receptors CD4 and CCR5 on the macrophage surface. Future experiments should define whether macrophage capture of HIV-1 requires sialylated viral envelope glycoproteins or depends on recognition of membrane gangliosides as observed for mDCs.

**Figure 2:**
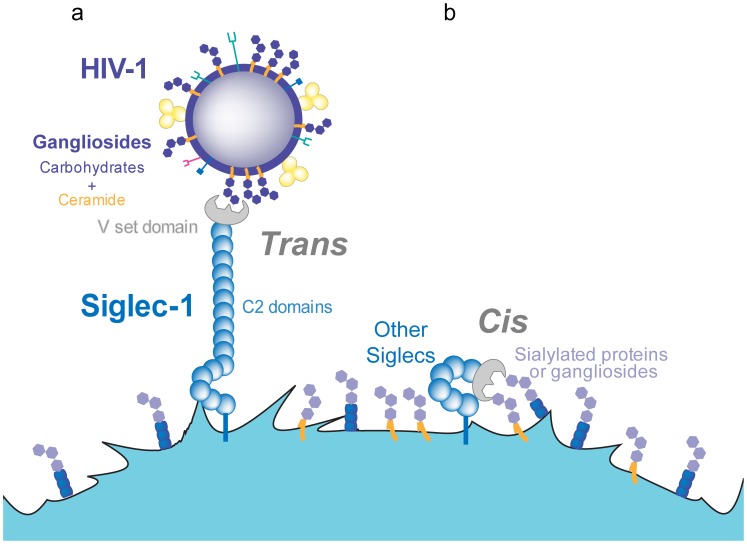
*Trans* and *cis* recognition of Siglec-1 ligands. **A.** Siglec-1 has 16 C2-type domains that extend the V-set domain from the glycocalix of the cell, allowing for recognition of sialylated molecules on different ligands and pathogens such as HIV-1. **B.** Other members of the Siglec family display a lower number of C2-type domains and interact only in *cis*, with sialylated molecules exposed on the membrane of the same cell.

Although all Siglecs have the potential to interact with sialylated gangliosides through their respective V-set domains, several distinctive features help to explain why Siglec-1 is the only family member that effectively mediates HIV-1 capture [66], [67], [69]. Siglec-1 is the largest member of the family, containing 16 Ig-like C2-type extracellular domains [70]. These domains separate the ligand-binding site from the cell surface, extending the V-set domain beyond the glycocalix of the cell (Figure 2). Thus, Siglec-1 is available for interaction with external ligands, while shorter Siglecs mainly bind cell-surface molecules in *cis* [65], masking their potential HIV-1 binding capacity (Figure 2). Siglec-1 constructs containing less than six Ig-like C2-type domains are unable to mediate sialic-acid-dependent binding in *trans* unless the cells are treated with sialidases to remove their own cell-surface sialic acids [71].

The affinity of Siglec-1 for sialylated ligands is in the micromolar range, but high-avidity binding can be achieved upon receptor and ligand clustering [65]. Live-cell imaging of viral capture by mDCs shows that viruses rapidly bind over the entire plasma membrane but subsequently traffic towards one pole of the cell, where they gradually accumulate and cluster [35]. Lipidomic analysis of HIV-1 membranes estimated that there are 12,000 GM3 molecules per virion [72], [73]. Other gangliosides such as GM1 are also present in HIV-1 membranes [58] but remain to be quantified. The presence of thousands of sialyllactose-containing gangliosides in the viral membrane is expected to support high-avidity interactions with the host-cell plasma membrane. These multiple interactions should yield stable viral attachment despite the relatively poor affinity of each individual interaction; the binding strength may thus be superior to that achieved by the higher affinity interaction of DC-SIGN or the viral CD4 receptor with only 14±7 envelope trimers per virion [74].

## Siglec-1 Role as a Pathogen Recognition Receptor

Siglec-1 binds promiscuously to many sialylated molecules typically found on pathogens, with a preference for N-Acetylneuraminic acid (Neu5Ac) in an α2–3 linkage [70]. This observation suggests that Siglec-1 may serve as a pathogen recognition receptor [65]. Lipidomic analyses of viral membranes revealed the presence of sialylated gangliosides in retroviruses, including HIV-1 and murine leukemia virus (MLV), and in vesicular stomatitis virus and Semliki forest virus [58], [73], [75]. MLV is also efficiently captured by mDCs via Siglec-1 [58], [67]. Siglec-1 may thus function as a general recognition receptor for many enveloped viruses, leading to viral uptake into mDCs and the induction of specific antiviral responses. Accordingly, Siglec-1-expressing myeloid cells efficiently capture VSV in vivo, either facilitating antiviral B cell responses or preventing viral neuroinvasion via type I interferon release [76], [77].

Exclusion of sialyllactose-containing gangliosides from viral budding domains or incorporation of neuraminidases that desialylate viral membrane glycolipids could interfere with mDC capture and immune recognition. Influenza virus is largely devoid of GM3 due to the viral neuraminidase [78], and it will be interesting to determine whether this feature is important for escape from immune recognition. HIV-1, on the other hand, appears to subvert this cellular recognition pathway for its own benefit. Indeed, enhanced HIV-1 capture by mDCs does not correlate with better viral antigen presentation ability; iDCs are capable of inducing higher antigen-specific T cell responses than mDCs [79].

Sialic acid is also present on the surface of several medically relevant nonviral human pathogens such as *Neisseria meningitidis*, *Haemophilus influenzae*, group B *Streptococcus*, *Campylobacter jejuni*, and several strains of *Escherichia coli*, as reviewed in [65]. These pathogens carry a lipopolysaccharide on their surface with an external moiety that is similar to the sugar moiety in human gangliosides [80]. Conceivably, these sialylated sugars may be recognized by mDCs and lead to pathogen capture and subsequent immune clearance. Recently, Siglec-1 has been shown to mediate uptake of sialylated *C. jejuni* in macrophages, promoting rapid proinflammatory cytokine secretion and type I interferon responses [81]. It will thus be interesting to determine whether these pathogens are also captured by mDCs via Siglec-1. Although mDCs markedly down-regulate their capacity for macropinocytosis, they are still able to capture, process, and present antigens internalized via endocytic receptors, suggesting that they may continuously initiate responses to newly encountered antigens during the course of infection [82], [83]. mDC capture of particles with ganglioside-containing membranes via Siglec-1 may have evolved as a mechanism for recognition of sialylated pathogens, and HIV-1 may have subverted this pathway for efficient viral spread.

The role of Siglec-1 in immune surveillance is further underscored by its ability to capture secreted exosomes and other microvesicles [66], [84], which are enriched in gangliosides [85]. Although their role in vivo is still controversial, exosomes are capable of transferring antigens to other target cells and thus could effectively increase the number of antigen-presenting cells (APCs) presenting a particular epitope at a given time, amplifying immune responses [86], [87]. Thus, exosome targeting to Siglec-1 on the surface of mDCs could favor specific triggering of immune responses by specialized APCs.

**Figure 3:**
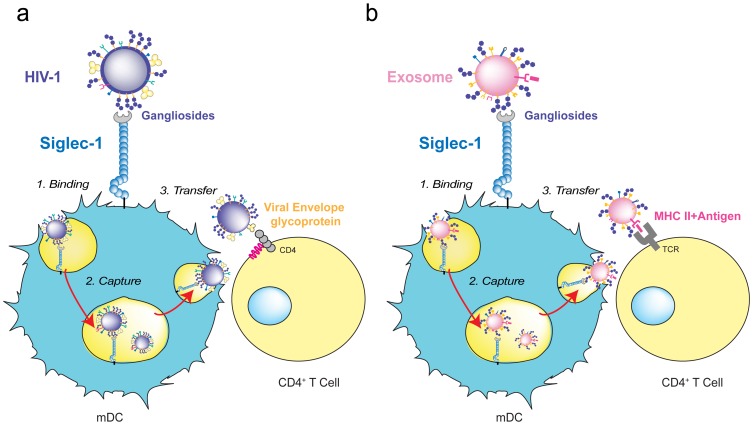
HIV-1 and exosome targeting to Siglec-1. **A.** HIV-1 binds to Siglec-1 through viral membrane gangliosides. Viral capture is followed by accumulation in a storage compartment until virus is released to infect a contacting CD4^+^ T cell via viral envelope glycoprotein and CD4/coreceptor interactions. **B.** Exosomes bearing processed antigens on MHC II molecules bind to Siglec-1 through recognition of their membrane gangliosides. They accumulate in the same storage compartment as HIV-1 until they are released and recognized by a CD4^+^ T cell via the interaction of the antigen-loaded MHC II on the exosome with an antigen-specific T-cell receptor on the target cell.

Intriguingly, captured exosomes do not need to be fully reprocessed and can induce immunity by direct release from mDCs, following a similar pathway as HIV-1 during *trans*-infection (Figure 3A). This occurs when captured exosomes expose previously processed functional epitope–MHC complexes on their surface that can be recognized by antigen-specific CD4^+^ T cells (Figure 3B) [88]. Hence, beyond its function as a recognition receptor for sialylated pathogens, the ability of Siglec-1 to capture exosomes could reflect a wider role of this molecule in amplifying immune responses.

## Siglec-1 in HIV-1 Pathogenesis

**Figure 4:**
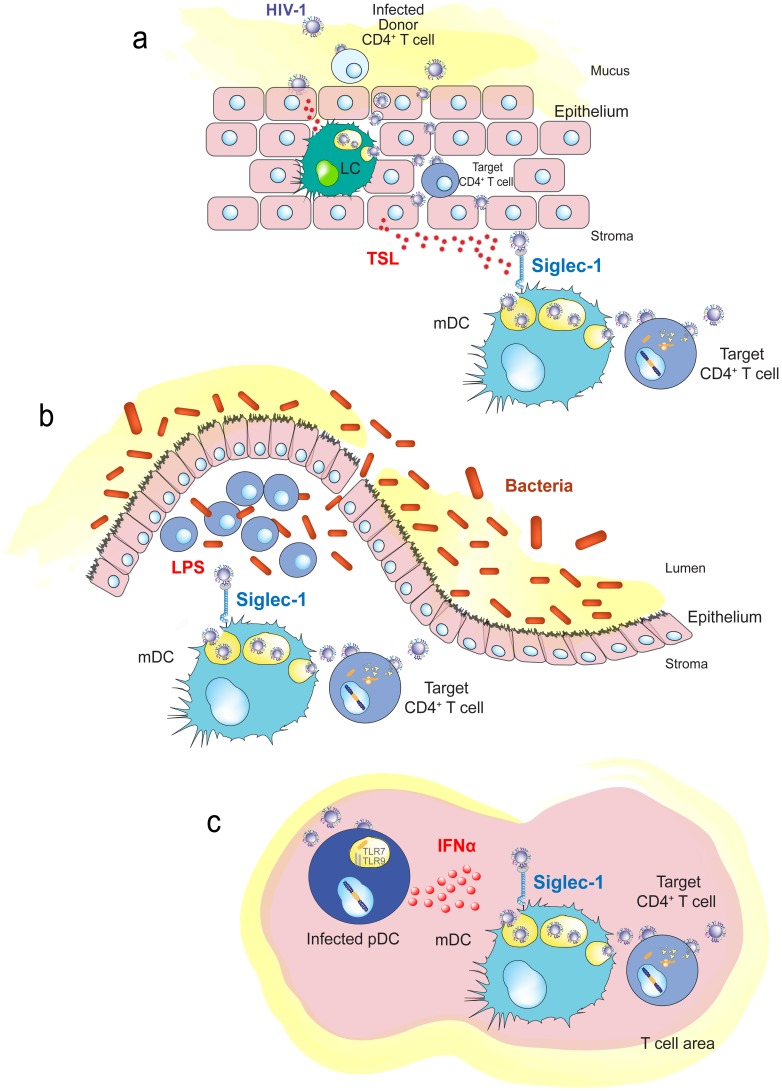
Immune activating signals can induce Siglec-1 expression and contribute to HIV-1 *trans*-infection. **A.** Human genital mucosal epithelial cells produce TSL in response to HIV-1. This cytokine could induce maturation of Langerhans cells or dermal DCs in the mucosa. **B.** Increased translocation of bacteria from the intestinal lumen after HIV-1 infection augments LPS levels that can stimulate DCs systemically. **C.** HIV-1-infected plasmacytoid DCs produce interferon α in lymphoid tissues, which triggers maturation of bystander DCs and induces Siglec-1 expression. Abbreviations: LC (Langerhans cells), pDC (plasmacytoid DC) TLR (toll-like receptor), TSL (thymic stromal lymphopoietin).

Upon HIV-1 exposure, human genital mucosal epithelial cells produce thymic stromal lymphopoietin (TSL) (Figure 4A), a secreted factor leading to maturation of DCs that also triggers DC-mediated amplification of HIV-1 infection in activated CD4^+^ T cells [89]. It will thus be important to address whether Siglec-1 mediates HIV-1 entry into these DCs or into vaginal Langerhans cells, where endocytosis of intact virions occurs primarily through a pathway independent of C-type lectin receptors [90]. Mucosal inflammation due to prior infection with other viruses, bacteria, or fungi can also stimulate the maturation of resident or incoming DCs by direct interaction with the invading pathogen or by secreted inflammatory cytokines and chemokines, favoring *trans*-infection events at early stages of HIV-1 invasion. This mechanism could partially explain why prior and concomitant sexually transmitted infections represent one of the strongest correlates of HIV-1 acquisition [91] and why blocking immune activation can protect animal models from mucosal simian immunodeficiency virus (SIV) transmission [92].

Chronic systemic immune activation is a hallmark of progressive HIV-1 infection, and various proinflammatory factors may induce Siglec-1 expression and contribute to HIV-1 *trans*-infection. This is the case for LPS, which is significantly augmented in chronically HIV-1-infected individuals, due to increased translocation of bacteria from the intestinal lumen [93]. The bacterial components may stimulate DCs systemically (Figure 4B), contributing to their maturation and therefore enhancing viral spread, while creating the proinflammatory milieu associated with chronic HIV-1 infection.

Interferon alpha (IFNα) is a potent antiviral cytokine produced by plasmacytoid DCs in response to HIV-1 challenge [94] that is also able to induce Siglec-1 expression in myeloid cells, such as DCs or monocytes [67], [69]. Thus, besides its antiviral function, IFNα can also favor HIV-1 *trans*-infection in an otherwise antiviral environment [67], [69]. Siglec-1 is up-regulated early after SIV infection in both monocytes from pathogenic and nonpathogenic animal SIV models, but its expression is only maintained in the pathogenic model [95]. Higher HIV-1 viral load in humans correlates with up-regulation of the Siglec-1 gene in circulating monocytes [96]. This could be orchestrated by plasmacytoid DCs (Figure 4C), which produce IFNα upon HIV-1 exposure and induce the maturation of bystander DCs [97].

## Concluding Remarks

Despite intensive research, there is uncertainty regarding the role of DCs in the establishment of HIV-1 infection in vivo. The field also lacks direct proof of DC participation in disease progression. The discovery of the role of Siglec-1 in capturing viruses with gangliosides in their membrane expands our understanding of HIV-1 transmission mechanisms and offers a new avenue to dissect the contribution of DCs to HIV-1 pathogenesis. In turn, this knowledge will help to design novel therapeutic approaches aimed to prevent viral dissemination.
